# ANALYSIS OF OHIO’S ASSISTED LIVING DIRECT CARE WORKFORCE

**DOI:** 10.1093/geroni/igad104.1098

**Published:** 2023-12-21

**Authors:** Katherine Kennedy, Cassandra Hua, Matt Nelson

**Affiliations:** Providence VA Medical Center, Providence, Rhode Island, United States; University of Massachusetts Lowell, Lowell, Massachusetts, United States; Miami University, Oxford, Ohio, United States

## Abstract

Direct care workers (DCWs) in Ohio’s licensed residential care facilities (RCFs) provide the most assistance with activities of daily living to individuals with disabilities. DCWs include medication aides, personal care aides, and nurse aides. In 2017, 41% of Ohio AL administrators reported high DCW recruitment issues and 36% high DCW retention problems (8+ on 10-point scale). Understanding how RCFs with no or little recruitment/retention challenges compare to other RCFs on strategies used, training hours, wages, benefits, resident composition, and RCF characteristics can identify potential strategies to increase recruitment/retention. This study uses the 2019 Ohio Biennial Survey of Long-Term Care Facilities (n=540 RCFs) to compare RCF strategies to recruit and retain DCWs by measures of recruitment/retention problem-severity and their 3-year retention rates among full-time (FT) and part-time (PT) DCWs. In 2019, 65% of Ohio RCF administrators reported recruitment issues at the median or greater (8+). Similarly, 62% of RCFs experienced retention issues at the median or more (7+). On average, 37% of FT DCWs worked in the RCF for 3 years or more and 21% among PT DCWs (ranges: 0%-100%). 28.24% of RCFs reported high 3-year retention among FT DCWs (60-100%) and 26.51% reported high 3-year retention among their PT DCWs (30%-100%). RCFs with less serious recruitment challenges report providing more hours of training for newly employed DCWs before they provide resident care (34.54 vs. 29.60, t=-2.7, p<.01). Individual retention strategies did not show relationships with high retention. Results indicate pervasive challenges with DCW retention and recruitment that require new approaches.

